# Effect of Parecoxib Sodium Preemptive Analgesia on the Recovery Period of General Anesthesia in Patients Undergoing Glioma Resection

**DOI:** 10.1155/2022/4934343

**Published:** 2022-06-13

**Authors:** Lijuan Zhu, Hui Guo, Tao Zheng, Jing Zhu

**Affiliations:** ^1^Department of Anesthesiology, Shaanxi Provincial People's Hospital, Xi'an 710068, Shaanxi, China; ^2^Department of Anesthesiology, Xi'an International Medical Center Hospital, Xi'an 710100, Shaanxi, China; ^3^Department of Neurosurgery, Xi'an International Medical Center Hospital, Xi'an 710100, Shaanxi, China

## Abstract

**Objective:**

To investigate the effect of parecoxib sodium preemptive analgesia on postoperative complications and postoperative recovery of patients undergoing glioma resection.

**Methods:**

A total of 200 eligible patients with low-grade gliomas in the functional brain area scheduled for an awake craniotomy between January 2017 and December 2020 were reviewed. The subjects were divided into two groups: the study group (*n* = 100) given dexmedetomidine plus parecoxib sodium for pre-emptive analgesia 30 minutes preoperatively, and the control group (*n* = 100) receiving dexmedetomidine alone. Venous blood was collected before surgery, at the time of postoperative recovery, and 24 hours after operation, mean artery pressure (MAP) and heart rate (HR) were recorded during surgery. Sedation satisfaction, agitation rate, numerical pain score (NRS), postoperative complications, minimental state examination (MMSE) scores, quality of life (QoL) scores, and incidence of adverse events were also investigated after the surgery.

**Results:**

There were no significant differences in operation time, awakening time, intraoperative awakening time, and extubation time between the two groups (*P* > 0.05). Compared with the control group, the ΔMAP (7.26 ± 2.21 versus 5.78 ± 2.36 mmHg) and the ΔHR (11.35 ± 3.66 versus 8.84 ± 2.47 beats/min) were significantly lower in the study group (*P* < 0.05). Compared with the control group, the satisfaction was higher and agitation rate was lower in the study group (*P* < 0.05). The incidence of intracranial infection and pulmonary infection decreased after operation (*P* < 0.05). The NRS of the study group was remarkably lower than the control group at 12 hours postoperatively Preoperative MMSE score and QoL score showed no statistical difference (*P* > 0.05), while postoperative MMSE and QoL scores showed statistical difference (*P* < 0.05).

**Conclusion:**

This study suggests that parecoxib sodium can significantly improve the level of sedation and analgesia in patients undergoing glioma resection, reduce the incidence of intracranial infection and pulmonary infection.

## 1. Introduction

Glioma is a malignant tumor originating from neurons and glial cells. It is the most common primary intracranial tumor, which accounts for 40%–50% of intracranial tumors [1]. Surgical resection is the most preferred treatment for glioma, which removes most of the tumor cells, effectively relieves patients' symptoms, and provides pathological support for further treatment [2]. Studies have shown that some neurosurgery patients have unconscious limb movements during anesthesia recovery and even self-removal of tracheal catheter, which in serious cases leads to catheter shedding, asphyxia, limb fracture, and increased perioperative wind risk [3]. Parecoxib sodium can reduce the afferent center of injurious stimulus, effectively reduce postoperative pain, and then reduce the incidence of postoperative agitation, which has important clinical value for patients to safely pass through the recovery period of anesthesia [4]. At present, the application of dexmedetomidine and parecoxib sodium in surgery has been reported in literature, but there are a few reports on the effects of the combined application of dexmedetomidine and parecoxib sodium on the anesthesia recovery period of neurosurgery patients. Therefore, this study investigated the effects of dexmedetomidine and parecoxib sodium, and the results are reported as follows [1].

## 2. Materials and Methods

### 2.1. Baseline Data

From January 2017 to December 2020, we conducted a retrospective analysis of 200 patients with low-grade glioma in the functional brain region (WHO I-II) in the neurosurgery department of our hospital.

### 2.2. Inclusion and Exclusion Criteria

#### 2.2.1. Inclusion Criteria

(1) Patients aged 35–70 years, a Karnofsky performance score (KPS) > 60 points; (2) patients with a confirmed diagnosis of glioma by clinical and MRI examinations, to receive an awake craniotomy for glioma resection; (3) patients with American Society of Anesthesiologists (ASA) grade I-II for anesthesia risk assessment; (4) patients undergoing craniotomy for the first time with no previous history of radiotherapy.

#### 2.2.2. Exclusion Criteria

(1) Patients with other malignant tumors; (2) known or suspected drug or alcohol abuse, and pregnancy; (3) allergy to NSAIDs or any of the drugs used in the study and coexisting diseases that could affect the reliability of assessments; (4) a history of significant cardiac, pulmonary, hepatic, or renal disease; (5) patients with mental disorders that prevented cooperation in completing intraoperative anesthesia assessment.

### 2.3. Anaesthesia and Analgesia

Patients in both groups underwent the same preoperative preparation, including routine monitoring of the electrocardiogram, pulse oximetry, invasive blood pressure, and bispectral index, and received awake craniotomy. A loading dose of 0.5 ug/kg dexmedetomidine was administered, anesthesia was inducted with propofol 2 mg/kg and remifentanil 0.1 *μ*g/(kg-min) that were administered intravenously, and rocuronium bromide 0.6 mg/kg was given via intravenous push. The lungs were ventilated mechanically with a tidal volume of 8–12 mL/kg at a frequency of 12–14 bpm to perform end-tidal carbon dioxide between 35 and 45 mmHg. Anesthesia was maintained with an effector compartment target concentration of 2.5 pg/ml at propofol target-controlled infusion, and remifentanil was infused at a rate of 0.1 ug/(kg-min). Patients in the study group were given 40 mg of parecoxib sodium intravenously for 30 min preoperatively and those of the control group were given the same dose of saline.

### 2.4. Data Collection

Demographic and intraoperative characteristics of patients such as age, sex distribution, weight, body mass index (BMI), operative time, recovery time, extubation time, and perioperative complications (including respiratory depression, nausea, vomiting, and shivering) were recorded. In addition, intraoperative wake-up time, and intraoperative range of MAP and HR were recorded (ΔMAP = MAP max–MAP min, ΔHP = HP max–HP min).

The Ramsay sedation score was used to assess the degree of intraoperative sedation during the wake-up period, and the numerical pain score (NRS) was used to assess the degree of wake-up analgesia of patients. Ramsay sedation score: 1 point: restless and irritable; 2 points: quiet and cooperative; 3 points: drowsy and able to follow instructions; 4 points: sleeping and arousable; 5 points: sluggish response to calling; 6 points: deep sleeping and unawakenable to calling. 2∼4 points were classified as satisfactory sedation, and 5∼6 points were classified as oversedation. The NRS scores at the time of postoperative recovery (T1), 2 hours postoperatively (T2), 12 hours postoperatively (T3), 24 hours postoperatively (T4), and 48 hours postoperatively (T5), were obtained by the patients circling the scores corresponding to the pain, where 0 points indicated no pain and 10 points indicated severe pain. Postoperative complications were recorded, including length of stay, 30-day readmission rate, intraoperative blood transfusion, and cardiopulmonary complications.

Intraoperative wake-up agitation was recorded and graded, in which grade 0 was quiet and cooperative; grade 1 was mild irritability, agitation, and intermittent moaning during stimuli; grade 2 was agitation and continuous moaning without stimuli, requiring immobilization of upper limbs; grade 3 was intensive struggling and agitation, attempting to extubate, and requiring immobilization on all limbs.

### 2.5. Postoperative Complications

The incidence of pulmonary and intracranial infections was significantly reduced with parecoxib, but the length of hospital stay, intraoperative blood transfusion, and other cardiopulmonary complications were not significantly different (*P* < 0.05).

### 2.6. Cognitive Function and Quality of Life Evaluation

The mini-mental state examination (MMSE) [5] scale was used to evaluate the cognitive function of the patients preoperatively and 1 month postoperatively. The MMSE scale consists of 11 items in five domains: orientation, memory, attention, language, and visual-spatial skills, with a total score of 30 points, and the higher the score, the better the cognitive function. The quality of life (QoL) scale [6] was used to assess the quality of life of patients preoperatively and 1 month postoperatively, and the QoL consisted of 12 domains, with a total of 5 points for each domain and a total of 60 points. The higher the score, the better the quality of life.

### 2.7. Statistical Analysis

All data analyses were performed using the SPSS 23.0 and visualized into required graphics via GraphPad Prism 9.0 software. The continuous variables with normal distribution were presented as mean and standard deviation, and the continuous variables with skewed distribution were presented as median (25^th^, 75^th^). Categorical variables were compared using *χ*2 test. Intergroup comparison was analyzed by repeated-measure analysis of variance. In all analyses, *P* < 0.05 was considered statistically significant.

## 3. Results

### 3.1. Demographic Characteristics of the Patients Included

A total of 200 patients were included for randomization. Baseline data of the 100 patients in the two groups are given in Table 1. All eligible patients showed similar baseline characteristics including age, gender, BMI, ASA classification, and WHO grading (*P* > 0.05) (Table 1).

### 3.2. Perioperative Indices

In the control group, the intraoperative wake-up duration was (30.45 ± 3.03) min, the ΔMAP was (7.26 ± 2.21) mmHg, and the ΔHR was (11.35 ± 3.66) times/min; in the study group, the above indicators were (29.42 ± 4.33) min, (5.78 ± 2.36) mmHg, and (8.84 ± 2.47) times/min, respectively. Similar intraoperative wake-up durations were observed in both the groups (*P* > 0.05). Dexmedetomidine plus pre-emptive analgesia with parecoxib sodium resulted in significantly milder changes in MAP and HR versus dexmedetomidine (*P* < 0.05) (Table 2). There was no significant difference in operation time, postoperative recovery time, and extubation time with parecoxib sodium (*P* > 0.05) (Table 3).

### 3.3. Sedation and Agitation in Intraoperative Wake-Up

In the control group, there were 13 cases of inadequate sedation and 5 cases of oversedation, with a sedation satisfaction rate of 82.00% (82/100); in the study group, there were 7 cases of inadequate sedation and 1 cases of oversedation, with a sedation satisfaction rate of 92.00% (92/100). The sedation satisfaction rate of the study group was higher than that of the control group, and the difference was statistically significant (Table 4).

In the control group, there were 88 cases of agitation grade 0, 3 cases of grade 1, 5 cases of grade 2, and 4 cases of grade 3, with an agitation rate of 12.00% (12/100); in the study group, there were 90 cases of agitation grade 0, 4 cases of grade 1, 3 cases of grade 2, and 3 cases of grade 3, with an agitation rate of 10.00% (10/100) (Table 5).

### 3.4. Postoperative Pain

**Figure 1:**
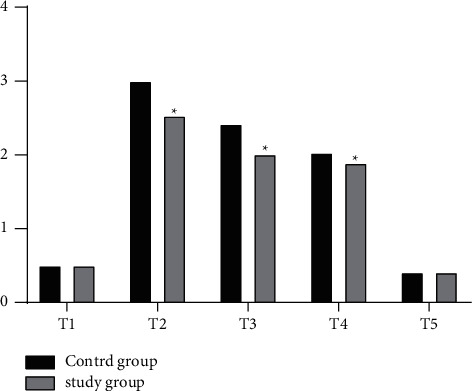
NRS scores.

After a slight increase in T2, the NRS scores steadily decreased from T3 to T4 in both the groups. The NRS scores of the study group were significantly lower than those of the control group at all time points from T2 to T4 (*P* < 0.05) (Figure 1).

### 3.5. Postoperative Complications

Postoperative complications is given in Table 6

#### 3.5.1. Cognitive Function and Quality of Life

Preoperative MMSE and QOL scores of the two groups showed no statistical difference. Compared with the control group, the value of MMSE decreased and the quality of life score increased after operation, with statistical significance (*P* < 0.05) (Table 7).

### 3.6. Perioperative Adverse Events

The study group had 5 cases of nausea and vomiting, and 5 cases of headache, with an incidence of adverse events of 10.00% (10/100). The control group had 6 cases of nausea and vomiting, and 3 cases of headache, with an incidence of adverse events of 9.00% (9/100). No new safety signals were identified. The two groups obtained a similar incidence of adverse events (*P* > 0.05) (Table 8).

## 4. Discussion

Neurosurgery usually takes a long time, requires a large amount of anesthetic drugs, and has a high incidence of restlessness during recovery from anesthesia. A strong stress response may increase blood pressure and heart rate, which may cause serious consequences and have a serious negative impact on perioperative safety [7]. Therefore, reducing stress response and agitation is of great clinical value to patients. In the review process, the performance of all eligible patients in intraoperative wake-up was favorable and the glioma resection was uneventful, which evidenced the promising analgesic effects of dexmedetomidine. In glioma resection, real-time patient feedback is essential to achieve the maximum excision of diseased tissue while preserving functional brain areas [8]. Agitation and inadequate sedation during intraoperative wake-up are associated with complications such as seizures, vomiting, and aspiration [9]. Cyclooxygenase (COX) is a key enzyme that catalyzes arachidonic acid (AA) to produce various prostaglandins (PGs). There are mainly two types of isoenzymes, namely COX-1 and COX-2 [10]. COX-1 is mainly involved in normal vasodilatory and contractile functions, gastric mucosal protection, gastrointestinal blood flow regulation, and maintenance of normal renal function [11]. COX-2 is an inducible enzyme, which usually exists at low levels in tissues but can be induced and produced in large quantities in the case of infection and injury. COX-2 is mainly expressed in neurons of cerebral cortex, hippocampus, amygdala, and spinal dorsal horn [12]. COX-2 expression was significantly increased in microglia and astrocytes under continuous neuroinflammatory response [13]. When inflammation occurs in the nervous system, COX-2 plays an important role in synaptic plasticity, neural development, learning and memory acquisition, and neurotransmitter transmission [14]. A large number of studies have shown that COX-2 expression are upregulated and play a key pathophysiological role in central nervous system diseases such as stroke, Alzheimer's disease, Parkinson's disease, multiple sclerosis, epilepsy, and schizophrenia [15].

Parecoxib sodium, a precursor drug of valdixib, is a COX-2 inhibitor that can reduce prostaglandin synthesis, inhibit central sensitization and sympathetic nervous activity, increase pain threshold, maintain hemodynamic stability, and enhance sedation and analgesia [16]. The results of this study showed that dexmedetomidine combined with parecoxib sodium significantly improved the alleviation of MAP and HR fluctuations, increased sedation satisfaction, and reduced the incidence of agitation compared with dexmedetomidine alone. At present, there are a few literature studies about the effects of the combined use of parecoxib sodium and dexmedetomidine on restlessness in patients during recovery. The results of this study suggest that the combined use of the two can further reduce the incidence of restlessness and play a synergic role.

The mechanism may be related to the inhibition of prostaglandin production by inhibiting coxidase in peripheral and central nervous system, thus reducing peripheral and central nervous system sensitization caused by harmful stimulus and reducing inflammatory response. Cui et al. showed that COX-2 was upregulated in glioma tissues [17]. As a COX-2 inhibitor, parecoxib sodium can rapidly recast immune molecules to enhance immune function by limiting COX-2-driven immune evasion [18]. This may account for the decrease in the lung and intracranial infections with parecoxib. Perfect immune function is an important condition for maintaining neural activity. Under pathological conditions, cellular inflammatory response will lead to upregulation of the expression of proinflammatory cytokines and proinflammatory enzymes, thus promoting the neuronal cell death caused by inflammation [19]. With parecoxib, the MMSE score was reduced, indicating improved cognitive function. The incidence of adverse events was similar in the two groups, indicating that parecoxib sodium had no inhibition on COX-1 and had little inhibition on gastrointestinal mucosa.

## 5. Conclusion

In conclusion, the above two anesthesia schemes can be safely used for glioma resection, dexmedetomidine combined with parecoxib sodium has a lower agitation score during awakening, mild changes in blood pressure and heart rate during intraoperative awakening, and a significant decrease in the incidence of complications of intracranial infection and pulmonary infection during postoperative hospitalization. Systematic and large-sample prospective studies on the long-term prognosis of glioma patients with the combination of these two drugs are still lacking. Therefore, on the basis of this study, the influence of two anesthesia schemes on the incidence of postoperative complications of other types of intracranial tumors can be further explored.

## Figures and Tables

**Table 1:** Baseline characteristics of the patients.

	Control group (*n* = 100)	Study group (*n* = 100)	*t*/*χ*^2^	*P*
Age ($\bar{x}$ ± *s*, years)	39.45 ± 7.04	40.26 ± 6.83	0.758	0.363
Gender			3.089	0.079
Male	69	57		
Female	31	43		
BMI (kg/m^2^)	21.83 ± 2.21	21.93 ± 3.81	1.017	0.327
ASA classification			2.426	0.119
I	42	53		
II	58	47		
WHO grading			0.184	0.668
I	59	56		
II	41	44		

**Table 2:** Intraoperative wake-up ($\bar{x}$ ± *s*).

	Intraoperative wake-up duration (min)	ΔMAP (mmHg)	ΔHR (times/min)
Control group (*n* = 100)	30.45 ± 3.03	7.21 ± 2.11	11.42 ± 3.66
Study group (*n* = 100)	29.42 ± 4.33	5.73 ± 2.26	8.81 ± 2.47
*t*	0.463	2.794	3.595
*P*	0.348	<0.001	<0.001

**Table 3:** Perioperative indices ($\bar{x}$ ± *s*, min).

	Operative time	Recovery time	Extubation time
Control group (*n* = 100)	305.64 ± 30.23	21.83 ± 2.36	22.54 ± 1.94
Study group (*n* = 100)	304.31 ± 32.36	20.03 ± 2.61	22.23 ± 1.21
*t*	1.122	0.324	0.287
*P*	0.367	0.850	0.780

**Table 4:** Sedation in intraoperative wake-up (*n* = 100, %).

	Satisfied sedation (2∼4)	Unsatisfied sedation		Sedation satisfaction rate (%)
		Inadequate sedation (1)	Oversedation (5∼6)	
Control group (*n* = 100)	82	13	5	82.00
Study group (*n* = 100)	92	7	1	92.00
*χ*2				4.421
*P*				0.036

**Table 5:** Agitation in intraoperative wake-up (*n* = 100, %).

	Agitation grading				Agitation rate (%)
	Grade 0	Grade 1	Grade 2	Grade 3	
Control group (*n* = 100)	88	3	5	4	12.00
Study group (*n* = 100)	90	4	3	3	10.00
*χ*2					0.204
*P*					0.651

**Table 6:** Postoperative complications.

	Control group(*n* = 100)	Study group (*n* = 100)	*t*/*χ*2	*PP*
The length of time(LOF)	10.3 ± 3.2	11.4±4.1	0.350	0.890
30-day readmission rate	11(11)	10(10)	0.053	0.818
Intraoperative blood transfusion	21(21)	19(19)	0.125	0.724
Hypertension	25(25)	23(23)	0.110	0.741
Low blood pressure	11(11)	12(12)	0.049	0.825
Arrhythmology	5(5)	6(6)	0.096	0.756
Cardiac insufficiency	3(3)	2(2)	0.000	1.000
Lung infection	17(17)	7(7)	4.735	0.030
Intracranial infection	25(25)	12(12)	5.604	0.018
Mortality within 30 days	2(2)	1(1)	0.000	1.000

**Table 7:** Cognitive function and quality of life scores.

	MMSE		QoL	
	Preoperative	Postoperation	Preoperative	Postoperation
Control group (*n* = 100)	14.21 ± 2.31	25.07 ± 2.12	30.25 ± 4.58	52.35 ± 4.58
Study group (*n* = 100)	13.02 ± 1.21	19.41 ± 2.53	30.32 ± 4.47	40.26 ± 4.62
*t*	0.439	8.073	0.071	12.044
*P*	0.321	≤0.001	0.703	≤0.001

**Table 8:** Adverse reaction.

	Nausea and vomiting	Headache	Incidence of adverse event (%)
Control group (*n* = 100)	5	5	10.00%
Study group (*n* = 100)	6	3	9.00%
*χ*2			0.058
*P*			0.809

## Data Availability

All data generated or analyzed during this study are included in this published article.
